# DSA-Based 2D Perfusion Measurements in Delayed Cerebral Ischemia to Estimate the Clinical Outcome in Patients with Aneurysmal Subarachnoid Hemorrhage: A Technical Feasibility Study

**DOI:** 10.3390/jcm12124135

**Published:** 2023-06-19

**Authors:** Sebastian R. Reder, Steffen Lückerath, Axel Neulen, Katja U. Beiser, Nils F. Grauhan, Ahmed E. Othman, Marc A. Brockmann, Carolin Brockmann, Andrea Kronfeld

**Affiliations:** 1 Department of Neuroradiology, University Medical Centre, Johannes Gutenberg University of Mainz, 55131 Mainz, Germany; sebastian.reder@unimedizin-mainz.de (S.R.R.); cbrockma@uni-mainz.de (C.B.); andrea.kronfeld@unimedizin-mainz.de (A.K.); 2 Department of Neurosurgery, University Medical Centre, Johannes Gutenberg University of Mainz, 55131 Mainz, Germany

**Keywords:** digital subtraction angiography, aneurysmal subarachnoid hemorrhage, delayed cerebral ischemia, vasospasm, perfusion, outcome prediction

## Abstract

(1) Background: To predict clinical outcomes in patients with aneurysmal subarachnoid hemorrhage (aSAH) and delayed cerebral ischemia (DCI) by assessment of the cerebral perfusion using a 2D perfusion angiography (2DPA) time–contrast agent (CA) concentration model. (2) Methods: Digital subtraction angiography (DSA) data sets of n = 26 subjects were acquired and post-processed focusing on changes in contrast density using a time–concentration model at three time points: (i) initial presentation with SAH (T0); (ii) vasospasm-associated acute clinical impairment (T1); and (iii) directly after endovascular treatment (T2) of SAH-associated large vessel vasospasm (LVV), which resulted in n = 78 data sets. Maximum slope (MS in SI/ms), time-to-peak (TTP in ms), and maximum amplitude of a CA bolus (dSI) were measured in brain parenchyma using regions of interest (ROIs). First, acquired parameters were standardized to the arterial input function (AIF) and then statistically analyzed as mean values. Additionally, data were clustered into two subsets consisting of patients with regredient or with stable/progredient symptoms (or Doppler signals) after endovascular treatment (n = 10 vs. n = 16). (3) Results: Perfusion parameters (MS, TTP, and dSI) differed significantly between T0 and T1 (*p* = 0.003 each). Significant changes between T1 and T2 were only detectable for MS (0.041 ± 0.016 vs. 0.059 ± 0.026; *p* = 0.011) in patients with regredient symptoms at T2 (0.04 ± 0.012 vs. 0.066 ± 0.031; *p* = 0.004). For dSI, there were significant differences between T0 and T2 (5095.8 ± 2541.9 vs. 3012.3 ± 968.3; *p* = 0.001), especially for those with stable symptoms at T2 (5685.4 ± 2967.2 vs. 3102.8 ± 1033.2; *p* = 0.02). Multiple linear regression analysis revealed that a) the difference in MS between T1 and T2 and b) patient’s age (R = 0.6; R^2^ = 0.34; *p* = 0.009) strongly predict the modified Rankin Scale (mRS) at discharge. (4) Conclusions: 2DPA allows the direct measurement of treatment effects in SAH associated DCI and may be used to predict outcomes in these critically ill patients.

## 1. Introduction

Aneurysmal SAH (aSAH) represents a specific type of hemorrhagic stroke that accounts for approximately 5% of all stroke cases in most Western countries [1]. Compared to other entities, aSAH occurs more often in younger patients with a mean age of 50–60 years [2,3]. While a relatively large number of patients survive the initial hemorrhage, up to 30% of these patients undergo neurological deterioration due to SAH-associated delayed cerebral ischemia (DCI) [4,5]. Although the pathophysiology of DCI is not completely understood [4] a complex combination of factors, such as large vessel vasospasm (LVV), microcirculatory dysfunction, cortical spreading depolarization, and inflammation, is considered to impact the development of DCI [4,6,7]. The complexity of pathophysiological interactions becomes apparent by the fact that treating isolated radiographic traceable cerebral LVV does not necessarily improve outcomes [8,9]. DCI is diagnosed clinically by the focal or global neurological deterioration of SAH patients after the exclusion of other causes. Current guidelines recommend induced hypertension in the case of DCI. In cases presenting refractory DCI with cerebral hypoperfusion and impeding cerebral infarctions, the off-label use of intra-arterial vasodilators such as nimodipine (IAN) and transluminal balloon angioplasty (PTA) is widely accepted as rescue therapies [5,10,11,12].

Nonetheless, complications associated with the mode of application and duration of therapy with calcium channel antagonists, such as nimodipine, need to be considered. Following an oral application of nimodipine alone, mild hypotension occurred in 39% of cases [13]. After IAN, Kapapa et al. (2021) observed pulmonary dysfunction in 12%, myocardial dysfunction in 1.5%, and systemic hypotension in 8% of cases [14]. Kieninger et al. (2017) examined the complication profile of long-term IAN during continuous nimodipine infusion over a period of 10.5 (±4.5) days [15]. They detected a significantly increased oxygen demand only on the second day after initiation and a corresponding increased requirement for noradrenaline starting from the second day, which remained relatively stable in the following days [15]. A case of takotsubo cardiomyopathy was reported, although it was already known before the treatment, and two patients required brief resuscitation [15]. Ott et al. (2014) also reported an increased incidence of pulmonary edema, which resulted in 25% of the patient cohort ending lethally in combination with myocardial dysfunction [16]. Weiss et al. (2022) demonstrated an association between increased cerebral oxygen saturation and reduction in target blood pressure of induced hypertension after IAN and suggested its implementation in clinical practice [5].

One aim of endovascular therapy in DCI is to improve cerebral perfusion in order to prevent cerebral ischemia. Classically, angiography is used to determine the degree of cerebral vasospasm by measuring vessel diameter [17]. The degree of vasospasm, however, does not necessarily correlate with the degree of cerebral hypoperfusion [17,18]. Therefore, DSA-based determination of cerebral perfusion before, during, and after PTA or intra-arterial infusion of vasodilating drugs (e.g., nimodipine) could not only affect treatment decisions, but may also be used to validate the efficacy of any therapeutic measure. Furthermore, it would allow the neurointerventionalist to compare different degrees of hypoperfusion in case of a follow-up angiography and may even avoid the necessity of an additional perfusion CT frequently performed after the intervention. In gross summary, in real time angiography (here: DSA) there are three contrast agent phases: (1) arterial, (2) parenchymatous and (3) venous. Previous studies evaluated different methods of organ perfusion measurement using various theoretical models, such as the “steepest slope model” [19], the “up-slope model” [20] and the “maximum slope model” [18]. In this retrospective study, we therefore set out to quantify brain parenchyma perfusion in DSA projections by analyzing the contrast agent concentration–time (or C(t)**-**) curves using a maximum slope model and, furthermore, investigated whether the acquired data can predict patient outcomes.

## 2. Materials and Methods

### 2.1. Study Population, In-/Exclusion Criteria, and Angiography Setting

Retrospectively, we included data from patients who were treated for untreated ruptured brain aneurysms with initial clinical presentation of SAH between January 2013 and December 2019. They were included when there were available data at the time point of initial SAH without treated aneurysms (T0), before (T1), and after endovascular treatment of SAH-induced LVV (T2). Only images acquired strictly laterally at an angle of ±90° (tolerance ±10°) could be analyzed by our program (Table 1). Data from 26 patients (20 women; 6 men) met the criteria for inclusion (Table 1), which resulted in 78 data sets. To prove whether it is possible to predict patients’ outcomes at discharge after endovascular treatment of SAH-induced LVV, we clustered the study population by (1) regredient and (2) idem or progredient symptoms/Doppler signals immediately after the endovascular treatment (group 1 and 2).

Every DSA data set was acquired at the same biplanar angiography unit (AXIOM: Philipps, Netherlands) by neuroradiologists with more than five years of experience in cerebrovascular angiography using the angiography unit’s standardized “cerebrovascular” DSA protocol: two frames per second, 80 kV and automatic dose rate control (min 15 mAS). Iodine containing CA (Ultravist^®^-300: 300 mg iodine/mL, Bayer Vital, Leverkusen, Germany) at a 9:1 proportion (9 mL CA; 1 mL sodium chloride) was used and manually applied at an average bolus rate of 5 mL/s. It was injected via a standard 6F (“French”) diagnostic catheter positioned in the extracranial ICA ipsilateral to the side of vasospasm treatment. Post-treatment DSA (T2) was acquired 34.8 ± 17 min after initial DSA and routinely performed approximately 5 min after therapies’ end to estimate treatment effects in the brain parenchyma.

### 2.2. Bolus Tracking Model

**Figure 1:**
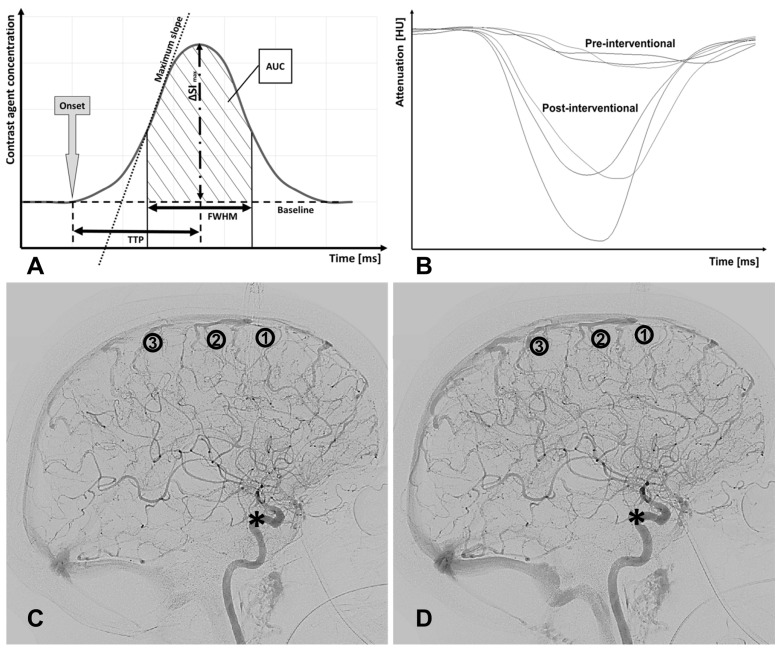
Bolus tracking model, its estimated parameters and positions of the three regions of interest (ROI) in lateral projection: (**A**) Schematic of the bolus tracking model with contrast agent (CA)–time curve (or C(t)-curve) and its estimated parameters with the onset, time to peak (TTP; time in ms from the onset until the CA bolus’ maximum), the full width of the half of the CA curve’s maximum (FWHM), the area under the curve (AUC), the maximum slope of the curve (MS in SI/ms), and the maximum amplitude of the CA bolus (ΔSI_max_ or dSI). (**B**) MatLab-based output function of the three ROIs at T1 (pre-interventional treatment) and T2 (post-interventional treatment). The algorithms were able to detect the CA-dependent attenuation of radiation, which is inverted to the CA concentration (and the C(t)-curve). (**C**) Brain parenchymogram (or minIP) before and (**D**) after i.a. nimodipine (IAN). The ROIs (1–3) were positioned to strictly measure brain parenchyma in order to prevent erroneous results from measurements of larger overlying arteries or veins. For calculation of the arterial input function (AIF), an ROI was positioned in projection on the cavernous segment of the internal carotid artery (ICA, marked by an asterisk) in order to normalize the results on the injection speed and blood flow.

An algorithm based on the C(t)-curve was programmed in MATLAB R2017b (The MathWorks, Inc., Natick, MA, USA) to analyze the DSA data (Figure 1A). DICOM data sets were read before vasospasms (T0), before (T1), and after interventional treatment (T2). In a lateral DSA projection, three regions of interest (ROI) with a predefined diameter of 40 pixels were drawn in the brain parenchyma on a minimum intensity projection (minIP) of the time course (see ROI 1 to 3 in Figure 1C,D). Greater cortical arteries and veins were not included in the ROIs, which could lead to incorrect perfusion values. The mean signal intensity of the ROI (or radiation attenuation) at each time point was subtracted by the baseline signal intensity and inverted to obtain C(t)-curves. To determine the arterial input function (AIF), an ROI with a diameter of 150 pixels was drawn covering the cavernous segment of the intracranial internal carotid artery (ICA; see Asterisk * in Figure 1C,D). Ten pixels within the AIF-ROI with the earliest and strongest grey value changes determined the AIF and a related standardized curve function (representative for the CA bolus passing by; exemplary at T1 and T2 see Figure 1B) [17]. As depicted in Table 1, several parameters were derived from the C(t)-curve and normalized through division by their corresponding value of the AIF (except for the onset—here, subtraction was performed):

- Onset (ms): first point in time of a significantly higher density (or CA concentration) compared to earlier time points.
- ΔSI_max_ or dSI: maximum difference in grey scales/maximum amplitude of CA concentration.
- Time to peak TTP (ms): time from onset to maximal signal attenuation/CA concentration (dSI).
- Maximum slope MS (SI/ms): maximal change in density (or CA concentration) per time, approximately corresponding with CBF [15,16].
- Full width at half maximum FWHM (ms): time interval between incoming and outflowing contrast agent bolus when half of the maximum difference in grey scales (dSI or ΔSI_max_) was reached.
- Area under the curve AUC: area enclosed by the C(t)-curve from onset to the endpoint of FWHM.

In more than half of the 78 DSA data sets (26 patients, DSA at three time points), the C(t)-curve was not acquired completely from the phase of arterial input to the end of parenchymal venous wash-out. A determination of a correct AUC or FWHM is impossible in these cases, so, primarily, MS, TTP, and dSI were analyzed.

### 2.3. Statistics

All analyses were performed using the statistical software SPSS (Version 29, IBM, New York, NY, USA). The different ROI-specific parameters (e.g., MS in ROI 1 to 3)were calculated and statistically analyzed as mean values (e.g., mean MS instead of analyzing MS for every single ROI). Due to a lack of normal distribution of parameters’ values (Shapiro–Wilk Test) and a small sample size (n < 30), non-parametric tests for related samples (Friedman Test) were used to determine differences from time point T0 to T2 [21,22]. Every result was corrected by the Bonferroni correction [23,24]. Intergroup differences between those patients with regredient symptoms immediately after interventional therapy of LVV and those without changes or progredient symptoms were analyzed by non-parametric Kruskal–Wallis Test [21,22]. Nominal scaled parameters were analyzed by using Fisher-corrected Chi-square Test [25,26]. Every *p*-value had a two-sided asymptotic significance niveau.

To predict patients’ outcomes at discharge, we defined three models for linear regression analysis. As coefficients, all models included the patient’s age and one different secondary coefficient. As a secondary coefficient, model 1 included MS at T1, model 2 MS at T2, and model 3 the difference in MS between T1 and T2. In Cohen’s power analysis, multiple linear regression analysis for predicting mRS at discharge needs a determination coefficient R^2^ = 0.297 to reach a high level of statistic power of 0.8 for n = 26, predictors = 2, and α = 0.05 (R^2^ = 0.358 for Power = 0.9) [27,28]. Multiple regression analysis showed R^2^ = 0.2 (model 1), R^2^ = 0.39 (model 2), and R^2^ = 0.335 (model 3). According to Cohen’s lowest estimation, model 1 had a medium effect, and model 2 and 3 had great effects [27].

## 3. Results

For retrospective analysis, 26 patients (20 women; 6 men) at the age of 50.26 ± 11.4 years met the study inclusion criteria (Table 1). As indicated in Table 2, most patients were severely affected (Fisher score 4; interquartile range (IQR) 1) and treated by i.a. nimodipine (n = 17). Most ruptured aneurysms were located in the anterior circulation (ACA; n = 13 respectively 50%), whereas the territory of the middle cerebral artery (MCA) was affected at most by vasospasms, either solely (n = 11) or in combination with other territories (with ACA n = 6; with PCA n = 1). mRS decreased from initial 5 (IQR 2) to 1 (IQR 3) in 26 patients within 3.1 (±1.9) years after release from hospital (Table 2). The study population was clustered by changes in post-interventional symptoms/Doppler signals for the analysis. Ten patients showed regredient symptoms or Doppler signals immediately after interventional treatment of LVV (group 1), whereas 16 had no relevant changes or progredient symptoms/Doppler signals (group 2). Between these groups, age, gender, Fisher score, Hunt and Hess score, localization and treatment of aneurysms, and days until vasospasm did not differ significantly. Group 1 had a significantly lower mRS at discharge (2.5, IQR 3.5 vs. 5, IQR 1 with *p* = 0.001), and at follow-up (1, IQR 1 vs. 4, IQR 4 with *p* = 0.012), approx. three years later than group 2 (2.5, IQR 3.5 vs. 5, IQR 1 with *p* = 0.001; 1, IQR 1 vs. 4, IQR 4 with *p* = 0.012). The localization of vasospasm differed pre-significantly: in group 1, the ACA territory was in 70% of cases affected by LVV, whereas in group 2, the MCA territory was affected in 75% (*p* = 0.07).

**Figure 2:**
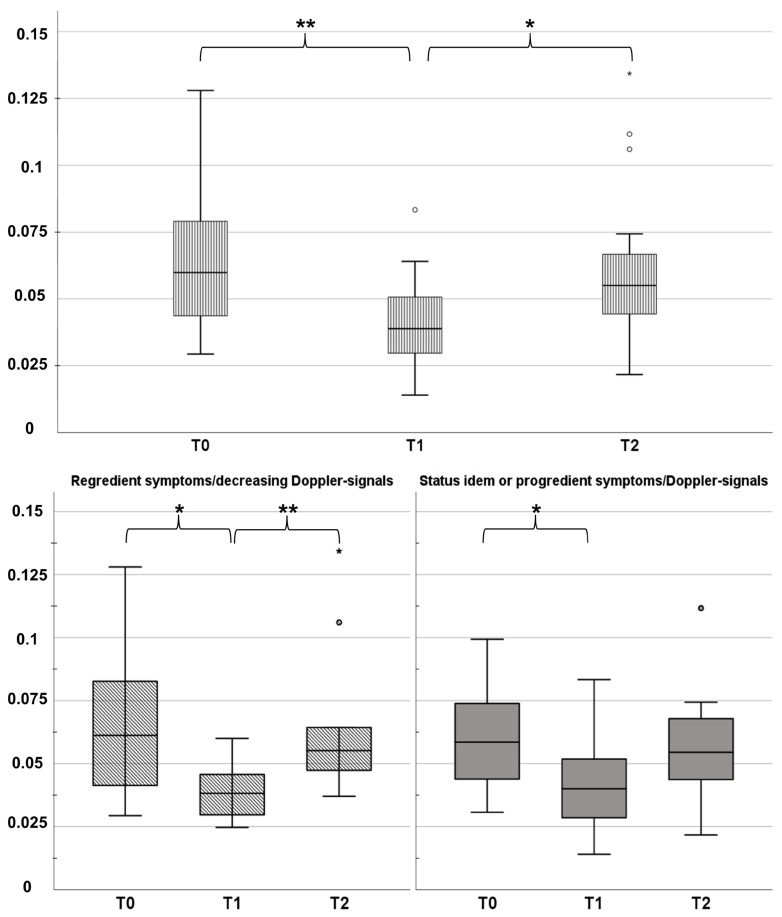
Maximum slope (**MS**) of the contrast agent bolus as mean over all ROIs. Changes in MS were clustered by the post-interventional course in regredient symptoms (group 1; overall *p* = 0.007) and status idem/progredient symptoms (group 2; overall *p* = 0.068). Abbreviated significance niveaus for sub-group analysis: * (0.05 > *p* > 0.01) and ** (0.01 > *p* > 0.001). Outlier values were standardized illustrated as small * in a boxplot column.

**Figure 3:**
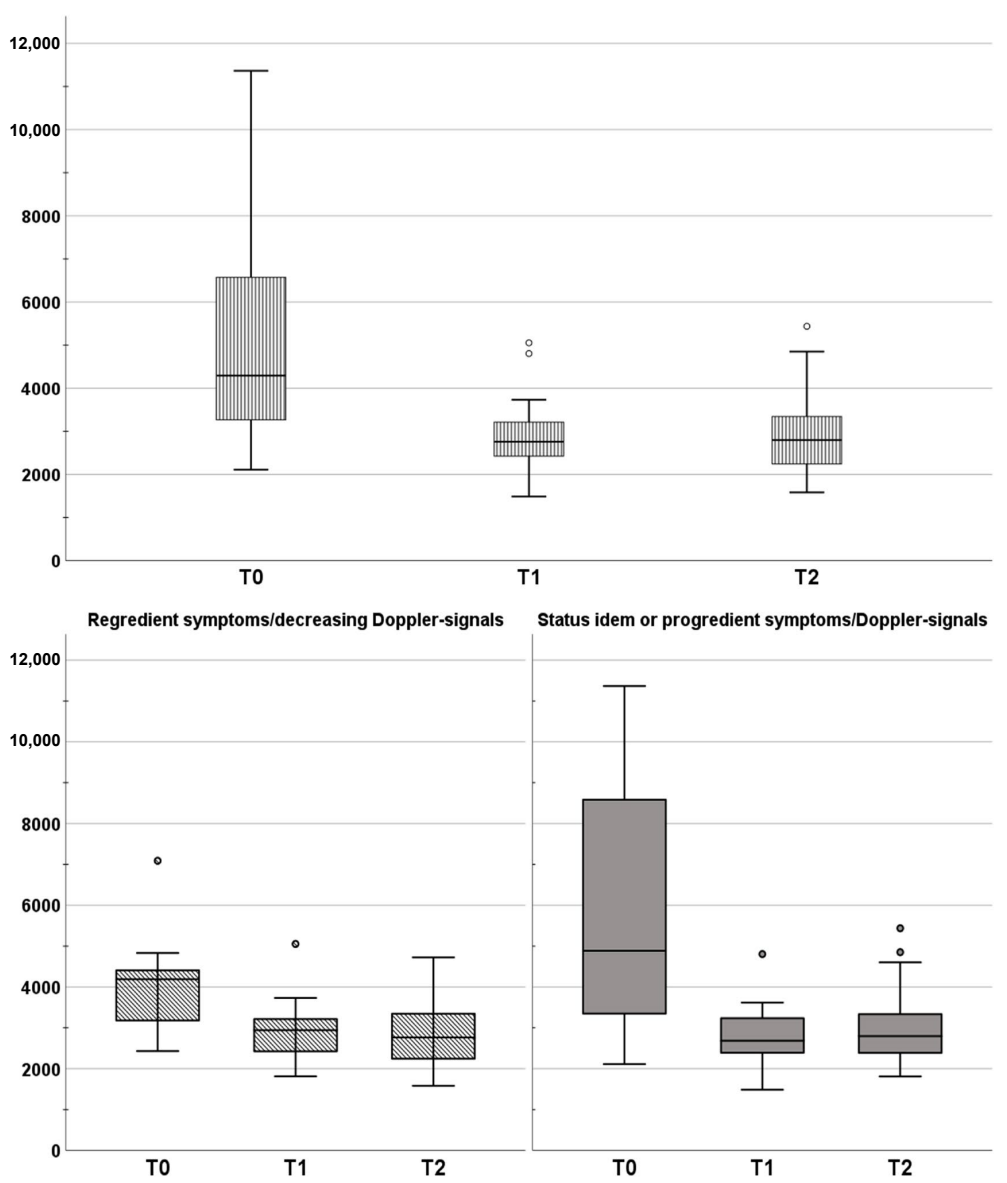
Maximum difference in the contrast agent bolus’ curve (**dSI**) as mean over all ROIs. Changes in dSI are clustered by the post-interventional course in regredient symptoms (group 1; overall *p* = 0.15) and status idem/progredient symptoms (group 2; overall *p* = 0.007).

**Figure 4:**
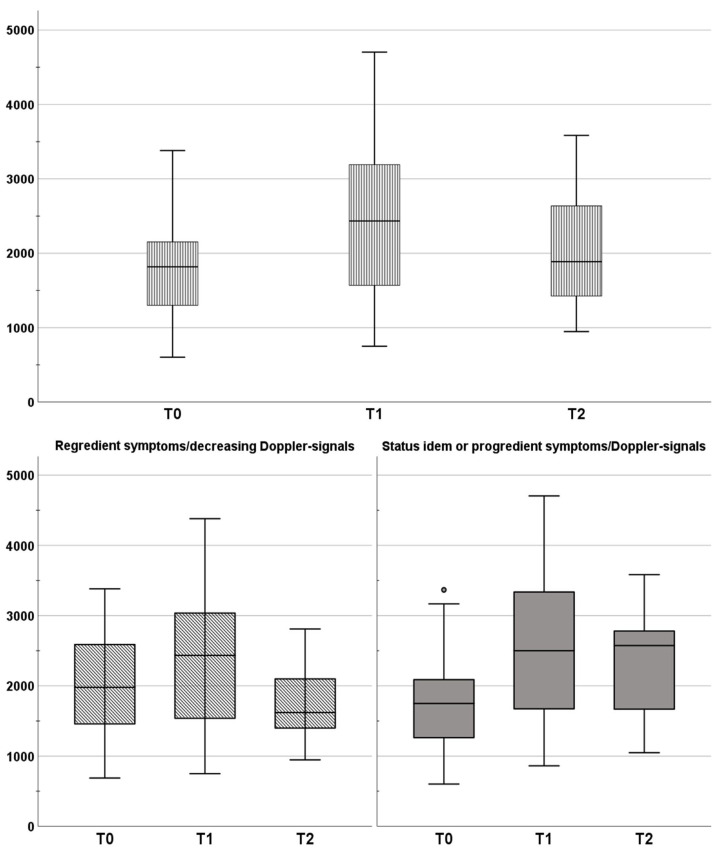
Time to peak (**TTP**) of the contrast agent bolus’ maximum as mean over all ROIs. Changes in TTP are clustered by the post-interventional course in regredient symptoms (group 1; overall *p* = 0.15) and status idem/progredient symptoms (group 2; overall *p* = 0.01).

As depicted in Figure 2, Figure 3 and Figure 4, from T0 to T1, the whole study population showed a significant decrease in MS (0.066 ± 0.032 vs. 0.041 ± 0.016; *p* = 0.003) and in dSI (5095.8 ± 2541.9 vs. 2884.4 ± 819.1; *p* = 0.003), whereas TTP increased (1855.4 ± 768.8 vs. 2512.5 ± 1138; *p* = 0.003). Those changes could be interpreted as the correlate for LVV (MS ↓; dSI ↓; TTP ↑). Likewise, this profile could be determined for group 2 (*p* = 0.003 to 0.022). In group 1, only MS was reduced significantly from T0 to T1 (*p* = 0.014). From T1 to T2 for the whole study population, there was only a significant increase in MS (0.041 ± 0.16 vs. 0.059 ± 0.26; *p* = 0.011; see Figure 2), which was congruent to changes in group 1 (*p* = 0.004; Figure 2). In group 2, there were no significant changes from T1 to T2.

In regression analysis, we can determine strong effects (R^2^ > 0.26) in predicting mRS at discharge using two coefficients: (1) patients’ age and (2) MS (differs between the models). Model 1 (age and MS at T1) was not interpreted in detail regarding its medium statistical power (R^2^ = 0.2). Model 2 included age and MS at time point T2 (MS_T2_) and showed a high statistical power (Power = 0.94; R^2^ = 0.39). Both coefficients were highly significant (*p* = 0.01 each). Model 3 was defined by age and the difference in MS between T1 and T2 (ΔMS = MS_T1_ − MS_T2_). Likewise, it had a high statistical power (Power = 0.87; R^2^ = 0.34), whereby each coefficient was significant (*p* = 0.015 and *p* = 0.04). So, according to the regression analysis Formula (1), expectable mRS at discharge could be calculated [28], exemplary for model 3 (see Table 3; ΔMS is negative):

mRS [discharge] = B × Age + B × (MS_T1_ − MS_T2_) + Constant (model) (1)

## 4. Discussion

Endovascular treatment of DCI is a procedure frequently performed in patients with SAH-related LVV. The efficacy of endovascular treatment of DCI hitherto has been reported in terms of an increase in vessel diameter, improved blood flow using duplex sonography, an increasing ptO2 fraction measured by intraparenchymal placed catheters, and improved clinical status of a patient or in follow-up CT Imaging using perfusion CT. Whereas all of these methods have their benefits and merits, a more direct method to analyze the effects of endovascular treatment regarding cerebral perfusion would be of interest. Consequently, a method to visualize and calculate flow curves in 2D angiography images has recently been proposed (e.g., “syngo iFlow”; Siemens Healthcare GmbH, Forchheim, Germany). Whereas this method allows visualization of a complete DSA run in a color-coded single image, it does not provide relevant information on perfusion to the brain parenchyma, which is characterized by the parenchyma blush in a DSA run. The aim of the underlying study, therefore, was to evaluate the effects of endovascular treatment on cerebral perfusion in patients with DCI using 2D DSA data sets using a time–concentration C(t)-model. On the one hand, we could define the process of developing DCI (T0 to T1) as a reduction (1) in the maximum slope of the contrast agent (CA) curve (MS) and (2) in the maximum concentration of the CA curve (dSI), as well as (3) an increase in the time to the maximum CA concentration (MS ↓; dSI ↓; TTP ↑). On the other hand, especially MS was feasible to assess significant changes in brain parenchyma density while CA bolus was passing by over time. In consequence, it was possible to assess the post-interventional treatment success of DCI when MS increased from T1 to T2 (in mean +45%; *p* = 0.01). This was consistent for those patients with regredient symptoms immediately after interventional treatment and not significant for those with stable or progredient symptoms (+65% vs. +34%; *p* = 0.004 vs. *p* > 0.05). In addition, mRS at discharge (mRS early), as well as mRS in the 3-year follow-up examination, differed significantly between both groups. Patients in group 1 (regredient post-interventional symptoms) presented with significantly lower mRS compared to group 2 (patients with post-interventionally stable or progredient symptoms) in early mRS (2.5I QR 3.5 vs. 5, IQR 0.75) and in follow-up mRS (1, IQR 0 vs. 4, IQR 4). In regression analysis, the effect of interventional treatment on mRS at discharge was strongly predictable by using (a) patients’ age and the difference in MS between T1 and T2 (model 3), or (b) age and MS at T2 (model 2). According to (a), this meant, the greater the difference in MS_T1−T2_, the lower the early mRS (CAVE: ΔMS = MS_T1−T2_ is negative!); and to (b), the greater MS_T2_, the lower the early mRS (CAVE: Regression coefficient B of MS_T2_ is negative!).

Recently, several previous studies have investigated different new techniques for brain perfusion assessment. In this context, the analysis of DSA image data poses a challenge as it lacks objective LVV or DCI evaluation. This may be due to the fact that it is not possible to reliably assess vessel diameters below 0.5 mm in the DSA [29]. Previous studies have suggested that LVV and DCI are able to arise independently and that DCI seems to be multifactorial [4,6,7,30,31,32]. Struffert et al. (2013) were able to map CBV distribution during DSA-based temporary balloon test occlusion using parametric color coding [33]. Based thereon, Gölitz et al. (2016) used parametric post-processing algorithms to quantify in vivo pre- and post-interventional TTP and MTT (“cerebral circulation time”) and were able to obtain significant results regarding the perfusion situation out of the DSA data sets [34]. In their study, the authors observed significant differences in “cerebral circulation time” (CCT) before and after IAN application due to LVV after SAH [34]. In addition, they compared post-interventional decreased circulation time with brain vessel diameters and revealed significant relationships [34]. Greater vessel lumen correlated with lower circulation time [34]. Regarding this study, we detected a significant increase in CA bolus MS, likewise, which leads to the assumption that cerebral blood flow was improved.

This study has several limitations. The data analysis was retrospective and involved a small number of 26 patients (f: 20; m: 6). In addition, cerebral perfusion data were not adjusted for individual differences, such as lifestyle and familial pre-existing diseases. Due to the retrospective aspect of this study, other parameters possibly affecting brain perfusion (such as blood pressure) could not be analyzed sufficiently. Patients undergoing clipping often did not receive digital subtraction angiography (DSA) prior to treatment, which may be due to the fact that surgical clipping, as an option in acute situations, is more readily available compared to endovascular coiling. This led to an increased exclusion of patients who underwent clipping. Investigator-dependent differences in the acquisition of DSA data could have led to irritations to our results. Differences in the application of CA or radiation dose were best possibly corrected by the normalization to AIF but still may influence the results. More quantitative evaluations using real CBF- and CBV-values would have been possible if a deconvolution method had been used. Unfortunately, the signal quality was not suitable for this. In more than half of the 78 DSA data sets (26 patients, DSA data at three time points), the C(t)-curve was not acquired until the end of the parenchymal venous wash-out of the contrast agent. Thus, it is possible that comparative analysis of TTP and dSI (and possibly other parameters) showed a false-negative due to errors in measuring the onset or baseline. Retrospectively, it was not possible to determine between those patients with post-interventional stable vs. those with progredient symptoms/Doppler signals due to inconsistent data acquisition. Thus, patients were categorized into group 1 if there were regredient symptoms immediately after intervention and/or Doppler signals, and into group 2 if there were stable/progredient symptoms. This did not allow for an accurate correlation of post-interventional results with Doppler signals or the determination of an additional group (e.g., only progredient or stable symptoms/Doppler signals). Moreover, it should be taken into account that the measuring of perfusion values was based on the lateral projection 2D DSA data. In contrast to CT or other tomographic imaging methods, this means that the evaluated C(t)-curve represented the cumulated signal from all the tissue along the radiation beam in a lateral projection. As a consequence, small pathological effects could be masked by superimposed signals. We minimized this effect by (1) a local contrast agent administration in ICA only; and (2) ROI-positioning, preferably at the brain’s apex due to measuring as thin brain parenchyma as possible in a lateral projection. Imaging post-processing was performed by a single observer, so that reader-based inter-individual differences could be unattended. However, the algorithms to determine the C(t)-curve and its parameters were programmed by an engineer experienced in the evaluation of perfusion imaging experiments, which should support the reliability of measurements. Furthermore, the usage of Fisher’s correction for Chi-square Tests or Bonferroni correction for Friedman Tests could have led to false-negative values due to their conservative testing algorithms. Possible therapy-based complications, which could have affected the mRS at discharge, were not taken into account. Nevertheless, it was possible to retrospectively determine the mRS reliably using the available data from nine years ago.

## 5. Conclusions

In conclusion, the quantification of 2D DSA-based perfusion of brain parenchyma allows primarily an immediate analysis of treatment response, which we found to strongly predict patient outcome (mRS) at discharge as well as at the 3-year follow-up. In addition, we were able to identify the reduction in MS and dSI and the increase in TTP as important correlates to the progress of developing DCI. Further analyses are required to underpin these results and their correlation with other modalities, such as intraparenchymal ptO2 measurements, which are of interest. 2DPA may also serve as a valuable method for (1) faster and facilitated CT-free stroke work-up in the angiography suite, (2) improved monitoring of therapeutic effects in the clinical routine, and (3) research of ischemic illness.

## Figures and Tables

**Table 1:** Patient inclusion and exclusion criteria.

Inclusion Criteria	Exclusion Criteria
Aneurysmal SAH with relevant vasospasm	Traumatic SAH
DSA data available for each of the three time points: initial DSA with symptomatic SAH (T0), DSA before (T1), and after endovascular treatment (T2)	Hemorrhage from arteriovenous malformation
Period of time between January 2013 and December 2019	Tumor-associated hemorrhage
	Motion artifacts
	Right/Left anterior oblique DSA projections <80° and/or >100°
	Patients without DSA data before vasospasms

**Table 2:** Study population characteristics clustered by post-interventional symptoms in two subgroups: group 1 (regredient) and group 2 (idem or progredient symptoms/Doppler signals). *p*-value refers to inter-subgroup differences. Values provided as mean (±1 standard deviation), total number of patients (%), or median (IQR). Localization of aneurysm and vasospasm is divided into ICA (Internal carotid artery), BA (Basilar artery), ACA (Anterior cerebral artery), MCA (Medial cerebral artery), PCA (Posterior cerebral artery), and global (>2 arteries).

Subgroups		Study Population	Group 1	Group 2	*p*-Value
**Number of cases**		26 (100%)	10 (38.5%)	16 (61.5%)	
**Age (years)**		50.26 ± 11.4	45.9 ± 15.2	53 ± 7.7	0.23
**Sex**	Female	20 (70%)	7 (70%)	13 (81%)	0.6
	Male	6 (30%)	3 (30%)	3 (19%)	
**Fisher Score (I–IV)**		4 (IQR 1)	4 (IQR 1)	4 (IQR 1)	0.9
**Hunt and Hess (0–5)**		2 (IQR 3)	2 (IQR 2.75)	2 (IQR 3)	0.6
**Localization of Aneurysm**	ICA	1 (3.8%)	0	1 (6.3%)	0.6
	ACA	13 (50%)	5 (50%)	8 (50%)	
	MCA	4 (15.4%)	1 (10%)	3 (18.8%)	
	BA	7 (26.9%)	4 (40%)	3 (18.8%)	
	MCA + BA	1 (3.8%)	0	1 (6.3%)	
**Treatment** **modality**	Clip	8 (30.8%)	2 (20%)	6 (37.5%)	0.4
	Coil	18 (69.2%)	8 (80%)	10 (62.5%)	
**Days until symptomatic vasospasm**		7 ± 2.2	6.8 ± 2.5	7 ± 3	0.8
**Localization of** **vasospasm**	ACA	6 (23.1%)	3 (30%)	3 (18.8%)	**0.07**
	MCA	11 (42.3%)	1 (10%)	10 (62.5%)	
	ACA + MCA	6 (23.1%)	4 (40%)	2 (12.5%)	
	PCA	1 (3.8%)	1 (10%)	0	
	PCA + MCA	1 (3.8%)	1 (10%)	0	
	Global	1 (3.8%)	0	1 (6.3%)	
**Treatment of** **Vasospasm**	PTA	4 (15.4%)	2 (20%)	2 (12.5%)	0.6
	IN	17 (65.4%)	7 (70%)	10 (62.5%)	
	PTA + IN	5 (19.2%)	1 (10%)	4 (25%)	
**mRS at discharge**		5 (IQR 2)	2.5 (IQR 3.5)	5 (IQR 0.75)	**0.001**
**mRS at follow up**		1 (IQR 3)	1 (IQR 0)	4 (IQR 4)	**0.012**
**Years until follow up**		3.1 ± 1.9	3.3 ± 2.2	2.9 ± 1.8	0.8

**Table 3:** Regression analysis to predict mRS at discharge by using age and the maximum slope (MS) at different time points as coefficients. Other parameters (such as Fisher score) were not able to reliably predict the mRS at discharge.

**Model 1**			
**Predicted Variable**	**Parameters**	**Coefficient 1**	**Coefficient 2**
		**Age**	**MS_T1_**
mRS at discharge	R	0.45	
	R^2^	0.2	
	**Sig. (Model)**	0.07	
	Constant (Model)	0.59	
	Regressions coefficient B	0.07	−4.39
	β (standardized)	0.45	−0.038
	**Sig. (Coefficients)**	**0.025**	0.84
	Power (1 − β)	0.56	
**Model 2**			
**Predicted Variable**	**Parameters**	**Coefficient 1**	**Coefficient 2**
		**Age**	**MS_T2_**
mRS at discharge	R	0.62	
	R^2^	0.39	
	**Sig. (Model)**	**0.004**	
	Constant (Model)	2.1	
	Regression coefficient B	0.072	−30.43
	β (standardized)	0.46	−0.43
	**Sig. (Coefficients)**	**0.01**	**0.014**
	Power (1 − β)	0.94	
**Model 3**			
**Predicted Variable**	**Parameters**	**Coefficient 1**	**Coefficient 2**
		**Age**	**ΔMS = MS_T1_ − MS_T2_**
mRS at discharge	R	0.579	
	R^2^	0.335	
	**Sig. (Model)**	**0.009**	
	Constant (Model)	0.859	
	Regression coefficient B	0.07	23.35
	β (standardized)	0.45	0.37
	**Sig. (Coefficients)**	**0.015**	**0.04**
	Power (1 − β)	0.87	

## Data Availability

Data are available at the Department of Neuroradiology (University Medical Mainz) and can be requested from the director (M.A. Brockmann, MD, MSc). Each request should be based on a scientific hypothesis and review by an ethical committee. Any request must be made in writing. Data will be saved for ten years after publishing (according to GCP guidelines).
